# Proficiency in lung ultrasound and perceived barriers to adoption among emergency physicians after a short pneumonia training programme: a multicentre observational study in Swiss emergency department

**DOI:** 10.1136/bmjopen-2026-116508

**Published:** 2026-09-08

**Authors:** Cécile Bessat, Alizé Erard, Alexia Roux, Thomas Brahier, Veronique Suttels, Roland Bingisser, Markus Schwendinger, Tim Bulaty, Yvan Fournier, Vincent Della Santa, Magali Pfeil, Dominique Schwab, Jörg D Leuppi, Nicolas Geigy, Stephan Steuer, Friedemann Roos, Michael Christ, Adriana Sirova, Alexandra Atzl, Fabian Napieralski, Damien Tagan, Werner C Albricht, Olivier Hugli, Noémie Boillat-Blanco, Elena Garcia

**Affiliations:** 1 Infectious Diseases, University Hospital of Lausanne, Lausanne, Switzerland; 2 University of Lausanne, Lausanne, Switzerland; 3 Emergency Department, University Hospital of Basel, Basel, Switzerland; 4 Emergency Department, Kantonsspital Baden AG, Baden, Switzerland; 5 Emergency Department, Intercantonal Hospital of Broye, Payerne, VD, Switzerland; 6 Emergency Department, Hospital of Neuchâtel, Neuchâtel, Switzerland; 7 Emergency Department, Hospital Riviera-Chablais, Rennaz, Switzerland; 8 Emergency Department and University Medicine, Cantonal Hospital Baselland, Liestal, Switzerland; 9 Medical Faculty, University of Basel, Basel, Switzerland; 10 Emergency Department, St Clarasspital, Basel, Switzerland; 11 Emergency Department, Cantonal Hospital of Lucerne, Lucerne, Switzerland; 12 Emergency Department, Cantonal Hospital St Gallen, St. Gallen, Switzerland; 13 Intensive Care Unit, Hospital Riviera-Chablais, Rennaz, Switzerland; 14 Division of Infectious Diseases, Infection Prevention & Travel Medicine, Cantonal Hospital St Gallen, St. Gallen, Switzerland; 15 Emergency Department, University Hospital of Lausanne, Lausanne, Switzerland

**Keywords:** ULTRASONOGRAPHY, Diagnostic Imaging, EDUCATION & TRAINING (see Medical Education & Training), Emergency Departments, INFECTIOUS DISEASES, Respiratory infections

## Abstract

**Abstract:**

**Objectives:**

Lung ultrasound (LUS) is accurate for diagnosing pneumonia in the emergency department (ED), but standard training is time-intensive, limiting its widespread implementation. We evaluated LUS proficiency for pneumonia diagnosis and perceived adoption barriers after a short training programme.

**Setting:**

This study was conducted in the frame of the PLUS-IS-LESS trial (Procalcitonin and Lung UltraSonography-based antibiotherapy in patients with Lower rESpiratory tract infection in Swiss Emergency Departments) (NCT05463406), a pragmatic stepped-wedge cluster-randomised clinical trial evaluating a clinical management algorithm combining LUS and procalcitonin to guide antibiotic use for lower respiratory tract infections (LRTIs) in 10 Swiss EDs.

**Participants:**

All medical supervisors (senior registrars and senior physicians) from the participating EDs were invited to go through the PLUS-IS-LESS LUS training programme and all those who completed the training programme were included in this study.

**Methods:**

The training programme included an e-learning course, followed by a half-day on-site training session with theory and hands-on practice. For proficiency evaluation, a validated structured assessment of LUS skills (LUS-OSAUS) was adapted into a 32-question online quiz and five bedside LUS examinations. Success was defined as achieving a score ≥80% on both the online quiz and supervised practical assessment. Success rates were compared between physicians according to their characteristics (age, sex, medical experience, previous use of ultrasound or LUS, linguistic region of work and type of hospital) using a χ² test. A 6-month follow-up survey identified factors associated with non-certification and barriers to the clinical use of LUS for managing LRTIs.

**Results:**

Of 122 trained physicians, 83 (68 %) completed both quiz and supervised LUS and 61 (50%) achieved certification. The most challenging items were pleural line assessment (83% success), recognition of consolidations (83%) and decision-making based on LUS findings (72%). Physicians <40 years had a higher success rate (p=0.009). Among those without complete certification, limited access to an ultrasound machine and low perceived added value of LUS were the main identified reasons. Lack of time was the most frequently reported barrier overall to LUS integration into ED workflows (77%).

**Conclusion:**

After receiving short training and focused proficiency testing, only half of physicians achieved certification, underscoring the challenges of broad LUS implementation. Limited time, equipment access and low perceived clinical value were key barriers, and integrating LUS findings into decision-making remained difficult. Ongoing support, supervision and protected time may be needed to enhance LUS adoption in EDs.

**Trial registration number:**

NCT05463406.

**STRENGTHS AND LIMITATIONS OF THIS STUDY**

- This multicentre study evaluated a standardised short lung ultrasound (LUS) training and certification programme across multiple emergency departments (EDs), enhancing generalisability within routine clinical practice.
- Proficiency was assessed using a structured, objective evaluation method adapted from the LUS-OSAUS (Lung Ultrasound Objective Structured Assessment of Technical Skills) framework, enabling robust assessment of theoretical knowledge and practical skills and demonstrating the feasibility of remote supervision.
- The sample size was sufficient to describe overall performance but limited the ability to perform adequately powered subgroup and centre-level comparisons.
- The certification threshold was based on expert consensus rather than empirically validated cut-off values, and long-term skill retention or clinical impact was not assessed and would require longitudinal follow-up.

## Introduction

 The combination of portability, absence of ionising radiation and high diagnostic accuracy make lung ultrasound (LUS) a valuable tool for assessing patients with respiratory symptoms in emergency departments (EDs).^1 2^ LUS demonstrates excellent sensitivity and specificity for the diagnosis of pneumonia, offering a reliable alternative to chest radiography and, in selected cases, even to CT.^3–5^ By improving diagnostic accuracy, LUS may help reduce unnecessary antimicrobial use in patients presenting with lower respiratory tract infections (LRTIs). However, the diagnostic accuracy of LUS is dependent on the operator’s level of training and experience. LUS certification programmes tend to be time-intensive and require significant clinical and educational commitments, which often lead to incomplete training and high dropout rates before certification. Moreover, the lack of accepted standards for proficiency assessment results in substantial heterogeneity in the structure, content and certification requirements of current LUS training programmes.^6^ For example, formal training programmes typically consist of a theoretical component of approximately 10–20 hours, complemented by a practical component requiring 25–50 supervised scans and up to 100 to achieve certification.^7–11^ The limited feasibility of most available training programmes underscores the need to determine whether adequate proficiency can be attained through shorter and more easily implementable training approaches. Only a few studies have reported that proficiency in LUS can be reached after approximately 5–10 supervised examinations.^12 13^

The PLUS-IS-LESS study (Procalcitonin and Lung UltraSonography-based antibiotherapy in patients with Lower rESpiratory tract infection in Swiss Emergency Departments) is a pragmatic, stepped-wedge cluster-randomised trial conducted across the EDs of 10 Swiss hospitals evaluating an intervention combining LUS and procalcitonin to guide antibiotic therapy in patients with LRTIs.^14^ As part of this trial, participating ED physicians completed a brief, structured LUS training programme focused on the diagnosis of pneumonia, consisting of a half-day programme and five supervised LUS examinations.

The primary objective of this study was to assess ED physicians’ proficiency in LUS for the diagnosis of pneumonia following completion of the PLUS-IS-LESS LUS training programme. Secondary objectives included feasibility of the short training approach, factors associated with non-completion of the certification process, physicians’ satisfaction, perceived educational needs and perceived barriers to the clinical implementation of LUS in the management of LRTIs. We expected that a short-structured training programme would enable the majority of participating physicians completing the certification process to achieve predefined proficiency criteria, but that logistical and implementation-related barriers would limit full uptake and integration into routine clinical practice.

## Methods

### Study setting and population

This study was embedded in the PLUS-IS-LESS study, a stepped-wedge cluster-randomised trial conducted between December 2022 and February 2025 in 10 EDs of 5 tertiary (Cantonal Hospital of Baden, Cantonal Hospital of Luzern, Cantonal Hospital of St. Gallen, University Hospital of Basel, University Hospital of Lausanne) and 5 regional hospitals (Hospital of Neuchâtel, Intercantonal Hospital of Broye, Hospital Riviera-Chablais, Cantonal Hospital Baselland and St. Claraspital Basel), located in the French-speaking and German-speaking regions of Switzerland. This trial was registered at ClinicalTrials.gov (NCT05463406) and its current status is completed. Details of the PLUS-IS-LESS trial design and protocol have already been published.^15^ The training and proficiency assessments were conducted between January 2023 and February 2025, approximately 1 month before each participating centre transitioned to the intervention phase of the trial; follow-up surveys were sent 6 months later. The PLUS-IS-LESS LUS training programme targeted all ED medical supervisors (senior registrars and senior physicians) from the participating centres. Participation depended partly on each centre’s ability to release physicians from clinical duties, resulting in variation in recruitment across sites. Physicians unable to attend the initial training session were offered a catch-up session. Only clinicians who completed the full training pathway, including the e-learning module, theoretical session and hands-on practical training, were included in the study. Physicians who did not complete all training components were excluded. The proficiency analysis was conducted among participants who completed the certification process including the written quiz and supervised practical examination, regardless of performance. As this was an observational ancillary study nested within the PLUS-IS-LESS trial, no formal sample size calculation was performed. The sample size was determined by the number of eligible medical supervisors participating in the training programme across the 10 participating EDs. All participants provided verbal informed consent prior to participation.

### The PLUS-IS-LESS LUS training programme

**Figure 1:**
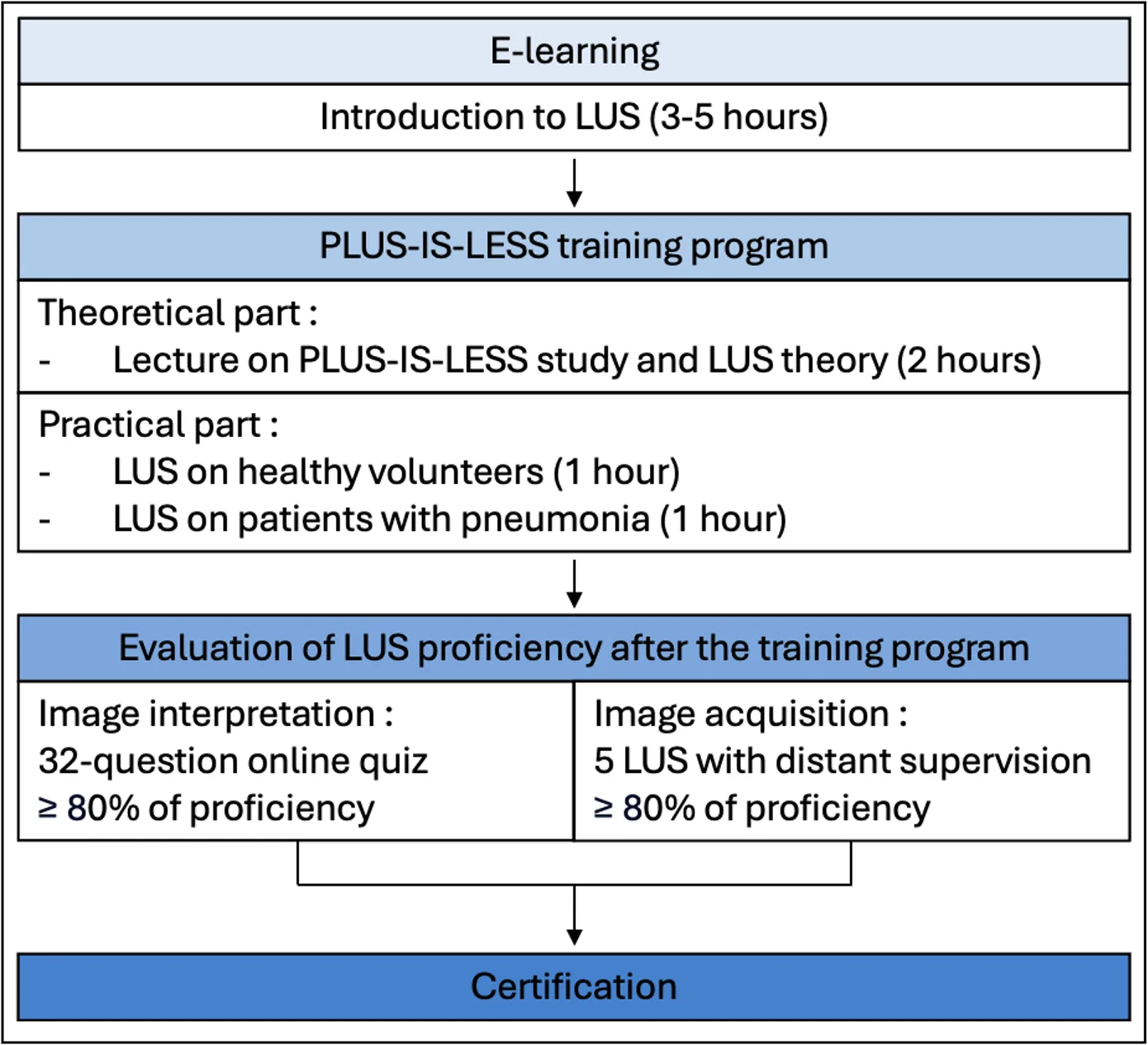
The PLUS-IS-LESS LUS training programme and certification process. LUS, lung ultrasound; PLUS-IS-LESS, Procalcitonin and Lung UltraSonography-based antibiotherapy in patients with Lower rESpiratory tract infection in Swiss Emergency Departments.

The PLUS-IS-LESS LUS training programme consisted of two sequential components: an e-learning course and a half-day on-site theoretical and practical session (figure 1). The e-learning course had an approximate duration of 3–5 hours and covered key LUS topics including basic principles, ultrasound findings, interstitial and pleural diseases, pneumothorax, consolidations and thoracentesis.^16^ The on-site training consisted of a 2-hour theoretical session reviewing LUS principles and ultrasound diagnostic patterns, followed by two 1-hour practical sessions: one on healthy volunteers and one on hospitalised patients diagnosed with pneumonia. Trainers were Swiss-certified LUS experts.

### Assessment of LUS image acquisition and interpretation proficiency

To assess post-training proficiency, a modified version of the Lung Ultrasound Objective Structured Assessment of Technical Skills (LUS-OSAUS) framework was used.^17 18^ Online supplemental appendix table A1 summarises the modifications to the original score that includes six areas—indication, systematic examination, technical skills, findings, documentation and clinical conclusion—distributed across 18 items. Proficiency was assessed through both theoretical and practical evaluations.

**Figure 2:**
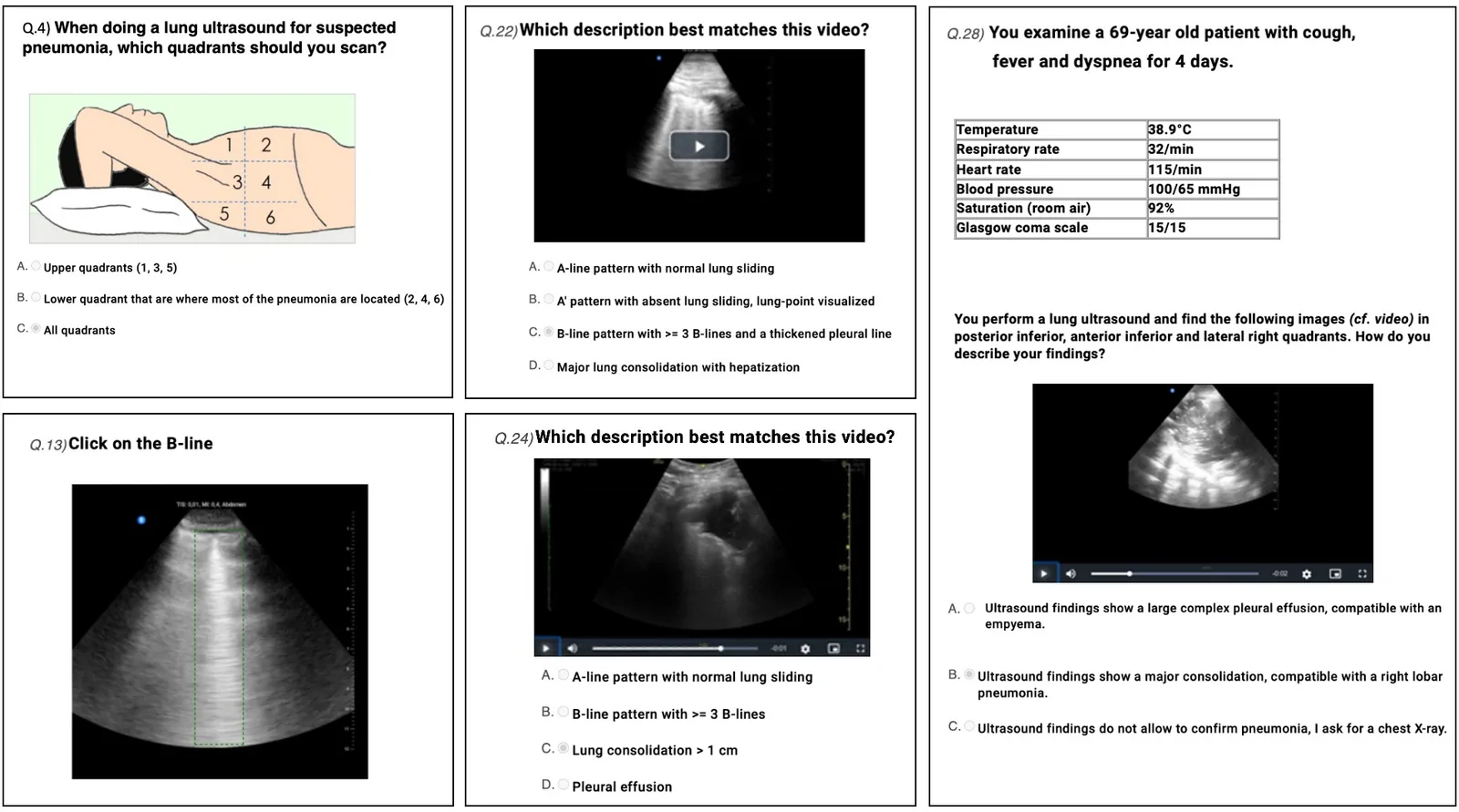
Example of online quiz questions based on our assessment score.

The theoretical assessment evaluated knowledge and image interpretation skills with a 32-question online quiz (ProProfs) (figure 2, full quiz available in online supplemental appendix table A2). Survey items were presented in a fixed order and were not randomised or alternated. The practical component assessed LUS examinations by trainees. Experienced LUS operators remotely reviewed the LUS scans based on predefined criteria for probe selection, preset, depth, gain, interpretability, saving and labelling. Participants without previous LUS experience were required to submit five LUS examinations, while certified experts submitted only two. Each examination followed a standardised LUS scanning protocol and included one image and one 5-second video per lung quadrant (12 quadrants total).

The full assessment pathway was considered completed once all four components had been fulfilled: the e-learning course, on-site training, online quiz and submission of the required LUS examinations. Certification required a score of at least 80% on the quiz and ≥80% of images rated as good quality in each LUS examination.

### Follow-up survey

Six months after their respective training programme, ED physicians were invited to complete an online follow-up survey via the ProProfs platform to assess barriers to completing the training and assessment process, provide feedback on the training format and identify obstacles to the implementation of LUS in clinical workflows. In case of non-response, multiple reminders, up to 12 months after their respective training were sent to increase participation. The survey included multiple choices, Likert scale and open-ended questions exploring opinions on the structure and content of the training, challenges encountered during the assessment process and on the clinical use of LUS and its integration into ED workflows. All survey questions are listed in the online supplemental appendix table A3.

### Statistical analysis

We calculated overall success rates for the quiz and the five LUS examinations, as well as success rates by each area and item, using simple proportions. Success rates were compared between ED physicians according to their characteristics: ultrasound use (regular or not), age (<40 years vs ≥40 years) and place of work (German-speaking or French-speaking region of Switzerland), using χ^2^ tests to explore factors associated with higher performance. No missing data were observed for the variables included in the analysis. Given the descriptive nature of the analyses and absence of major assumptions or missing data, sensitivity analyses were not performed. All statistical analyses were performed using R software (V.2023.06.2+561).

### Patient and public involvement

Participants were informed of study progress through newsletters and received overviews of personal results after tests at each time point.

## Results

### Participants

**Figure 3:**
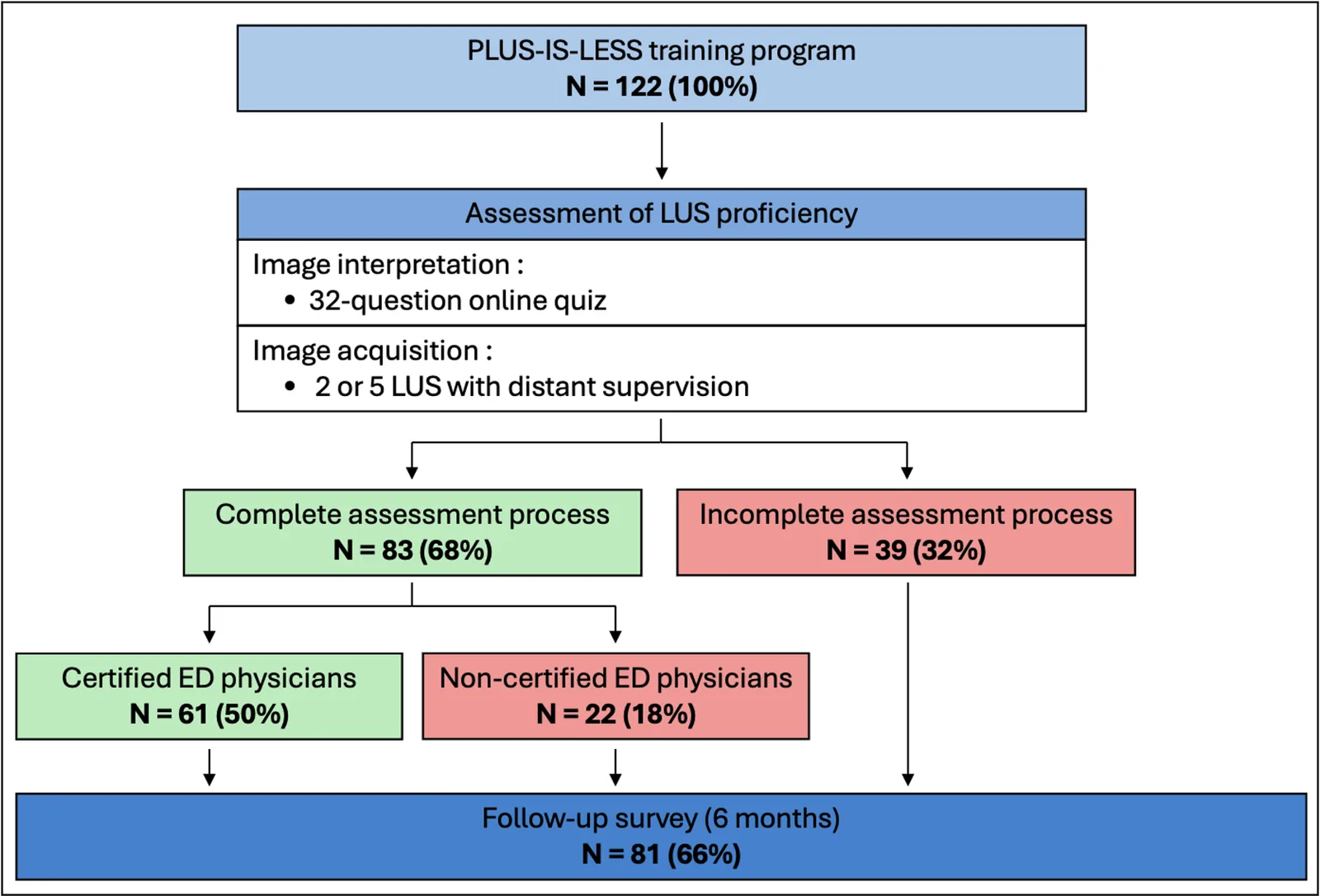
Flow chart of participants. ED, emergency department; LUS, lung ultrasound; PLUS-IS-LESS, Procalcitonin and Lung UltraSonography-based antibiotherapy in patients with Lower rESpiratory tract infection in Swiss Emergency Departments.

A total of 122 ED physicians participated in the LUS training programme. Among them, 112 (92%) completed the online quiz, and 83 (68%) completed both the quiz and the required LUS examinations; these participants were included in the proficiency analysis. The remaining 39 (32%) ED physicians were classified as having an incomplete assessment, meaning they did not complete the online quiz, the required LUS examinations or both. Ultimately, 61 (50%) of the participating physicians fulfilled all requirements necessary for certification. The follow-up survey was sent to all 122 physicians and completed by 81 (66%). Figure 3 presents the participant flow chart.

The characteristics of physicians included in the proficiency analysis are summarised in table 1. Overall, 28 (34%) were female and 48 (58%) were younger than 40 years. While 51% reported regular use of ultrasound in daily practice and 33% were already certified by an ultrasound society, experience with LUS was more limited, with only 36% using it regularly and 11% already certified in LUS. Physicians were evenly distributed between French-speaking and German-speaking regions (52% vs 48%), and between secondary and tertiary hospitals (48% vs 52%).

**Table 1:** Characteristics of ED physicians who completed the full assessment process

Category	n (%) of ED physicians (N=83)
Age (years)	
<40	48 (58%)
≥40	35 (42%)
Gender	
Female	28 (34%)
Male	55 (66%)
Medical experience (years)	
<5	8 (9.6%)
5–9	24 (29%)
10–14	24 (29%)
≥15	27 (33%)
Ultrasound experience	
Never performed ultrasound	0 (0%)
Few ultrasounds performed	14 (17%)
Regular ultrasound use	42 (51%)
Certified ultrasonographer	27 (33%)
Lung ultrasound experience	
Never performed lung ultrasound	3 (3.6%)
Few lung ultrasounds performed	41 (49%)
Regular lung ultrasound use	30 (36%)
Certified lung ultrasonographer	9 (11%)
Language region	
French-speaking	43 (52%)
German-speaking	40 (48%)
Hospital type	
Secondary (regional)	40 (48%)
Tertiary (university)	43 (52%)

ED, emergency department.

### Success rate

Of the 122 trained physicians, 83 completed both theoretical and practical assessments and are included in the proficiency analysis. Of those, 71 (86%) passed the theoretical assessment and 72 (87%) the practical examinations, resulting in an overall certification rate of 73%, with 61 physicians fulfilling both requirements. The median time from training to certification was 42 days (IQR 24–66).

Table 2 presents success rates by area and item according to the adapted assessment score. Among the six areas, the highest success rates were observed for systematic LUS examination (100%), technical skills (94%), documentation (94%) and indication (92%). A moderate success rate was noted in the findings area (86%), whereas clinical conclusion was the most challenging (72%). Among the 18 items, most achieved high success rates (≥90%). Moderate success rates were observed for pleura assessment (83%), consolidations assessment (83%) and depth adjustment (81%), and the most difficult item was the ability to establish a diagnosis based on LUS findings (72%).

**Table 2:** Success rates by area and item based on the adapted lung ultrasound assessment score

Areas and *items*	n (%) of ED physicians (N=83)
Area: Indication	76 (92%)
*Indication*	76 (92%)
Area: Systematic lung ultrasound examination	83 (100%)
*Systematic lung ultrasound examination*	83 (100%)
Area: Technical skills	78 (94%)
*Correct choice of probe*	80 (96%)
*Correct choice of preset*	81 (98%)
*Correct depth*	67 (81%)
*Correct gain*	79 (95%)
*Interpretability of the images*	78 (94%)
*Saving images/videos correctly*	78 (94%)
*Labelling anatomical position correctly*	80 (96%)
Area: Findings	71 (86%)
*Correct assessment of pleura*	69 (83%)
*Correct assessment of B-lines*	83 (100%)
*Correct assessment of consolidations*	69 (83%)
*Correct assessment of pleural effusion*	79 (95%)
*Correct assessment of diaphragm*	81 (98%)
*Correct assessment of M-mode*	79 (95%)
*Correct assessment of whether ultrasound-guided thoracentesis is safe*	82 (99%)
Area: Documentation	78 (94%)
*Documenting findings in patient’s chart*	78 (94%)
Area: Conclusion	60 (72%)
*Able to make a diagnosis on the basis of lung ultrasound findings*	60 (72%)

ED, emergency department.

**Figure 4:**
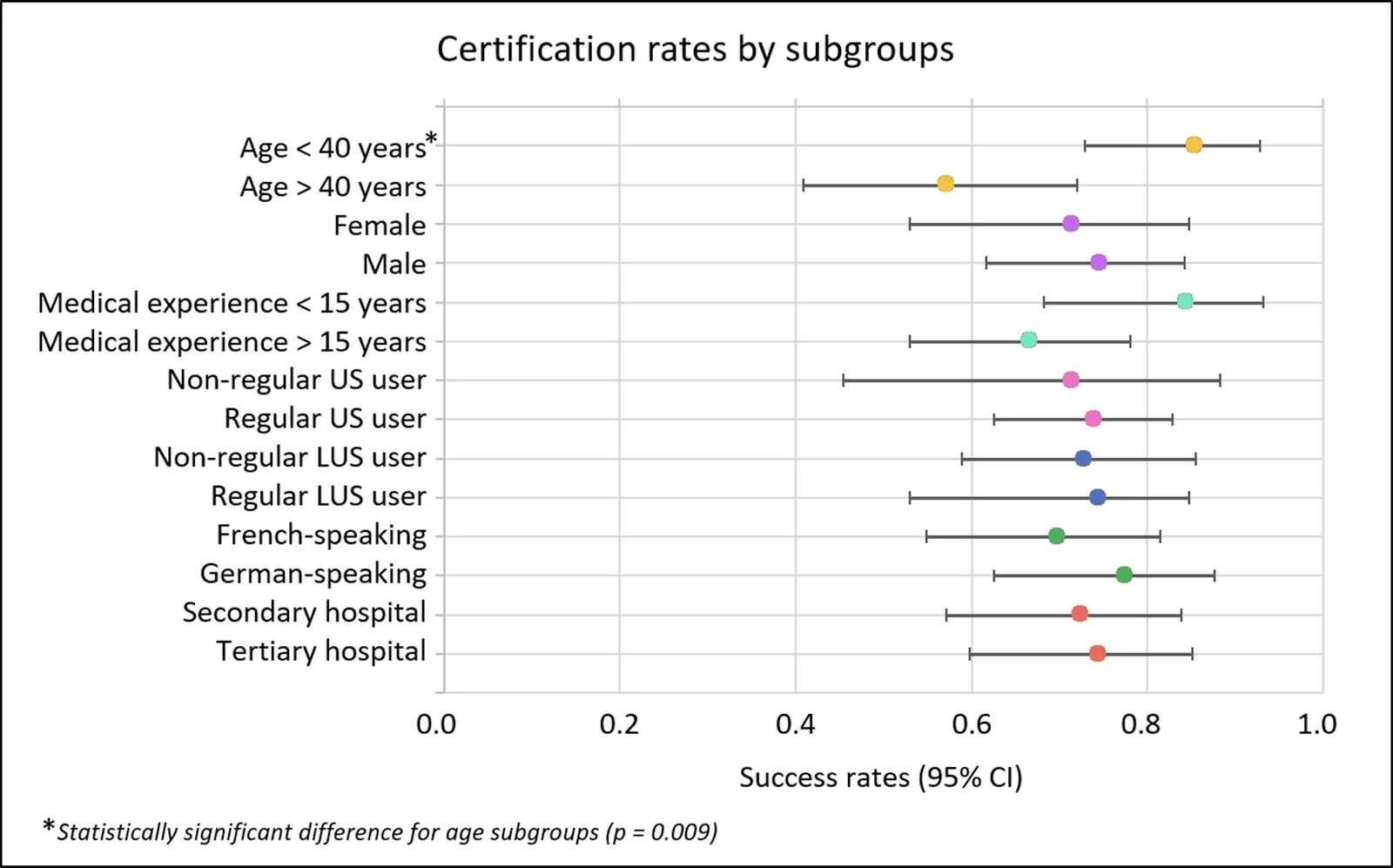
Certification rates by subgroups. LUS, lung ultrasound; US, ultrasound.

As shown in figure 4, the only factor significantly associated with certification was the age of the physician under 40 years (p=0.009). In this group, the certification rate was 85% (95% CI 0.73% to 0.93%), compared with 57% (95% CI 0.41% to 0.72%) among ED physicians aged 40 years or older. No other factors were significantly associated with higher certification rates. Detailed success rates and p value for all subgroups are available in online supplemental appendix table A4.

### Follow-up survey

Among the 122 ED physicians, 81 (66%) responded to the follow-up survey and were included in the analysis (figure 3), including 16 who were either non-certified or had not completed the full assessment process. In this subgroup of 16 ED physicians, the most frequently reported barriers to completing the training and assessment process were lack of time (81%), unavailability of an ultrasound machine (100%) and a perceived lack of added value of LUS in their clinical practice (100%).

Regarding perceptions of the training format, 61% of respondents reported feeling comfortable acquiring LUS images after performing 6–15 supervised examinations. Despite this, the majority (67%) considered the requirement of five LUS examinations to be appropriate for the study assessment process.

Several challenges were reported regarding the clinical integration of LUS across all ED physicians (certified, non-certified and those with incomplete assessment process) and could be grouped into patient-related, technical and organisational factors. Patient-related difficulties included obesity (82%) and patient weakness or poor cooperation (68%), particularly when patients were too frail or agitated to sit up or change position, making posterior lung scanning more cumbersome and often requiring additional staff. Technical challenges were mainly interference from the heart in the L2 quadrant (58%). Organisational and workflow-related barriers were overall lack of time (77%) with difficult integration into emergency flow.

In terms of continuing education, the most commonly reported needs were access to shared collections of pathological images (59%), protected time for supervised hands-on practice (52%) and opportunities for on-site supervision (44%). Full survey results are presented in online supplemental appendix A5.

## Discussion

When the training was brief and the certification requirements limited, only half of the physicians ultimately achieved certification, underscoring the challenges of implementing LUS training in high-workload settings. Nevertheless, among those who completed all certification requirements, the majority achieved high scores in theoretical knowledge and practical skills, indicating that short educational interventions can substantially enhance LUS proficiency. Younger ED physicians (<40 years) were more likely to achieve certification, possibly reflecting earlier exposure to Point of care ultrasound (POCUS), greater familiarity with digital tools or higher motivation to adopt novel diagnostic methods.

At the technical level, the most frequent error was suboptimal depth adjustment, although it rarely compromised image interpretability, and has been previously reported in the literature.^19^ Difficulties in identifying pleural lines and consolidations likely reflected overinterpretation of normal variants, consistent with prior studies.^20^ These findings support the need for targeted teaching on differentiating normal from pathological images and for structured feedback to strengthen image interpretation skills. Participants expressed the need for shared image libraries, follow-up sessions and protected time for supervised practice strategies associated with improved retention, diagnostic accuracy and confidence in POCUS use.^21–23^

Practical barriers raised such as limited time and restricted access to ultrasound equipment were frequently reported and must also be considered within the broader context of ED workflow and institutional organisation. In busy ED settings, high patient turnover, competing clinical priorities and pressure for rapid decision-making may reduce clinicians’ willingness or ability to incorporate LUS into routine assessments, particularly when the examination is perceived as time-consuming or logistically cumbersome. Limited equipment availability may further delay access and reduce opportunities for regular practice, potentially hindering skill consolidation and sustained uptake. These findings suggest that successful LUS implementation requires not only individual training but also institutional strategies that support workflow integration, adequate resource allocation and prioritisation of bedside ultrasound within routine emergency care pathways.^24^

Despite high success rate across most assessment areas, integrating LUS findings into clinical decision-making remained challenging. The ‘clinical conclusion’ area yielded the lowest scores, suggesting that translating sonographic findings into diagnostic reasoning and therapeutic decisions develops more slowly than image acquisition and basic interpretation.^24 25^ This indicates that barriers to implementation extend beyond image acquisition skills alone. Potential contributing factors may include uncertainty in incorporating ultrasound findings into diagnostic reasoning, limited confidence in interpreting findings within complex clinical presentations, time pressure in busy emergency department workflows and insufficient senior supervision or reinforcement in routine practice. In addition, clinicians may perceive limited added value of LUS if its impact on management decisions is not immediately evident in daily care. These findings highlight that successful implementation of LUS may require not only technical skills training but also educational strategies specifically targeting clinical reasoning, supervised decision-making and workflow integration.

Although initial participation was high, 32% of physicians did not complete the full certification pathway, underscoring a critical loss during the assessment phase. The follow-up survey indicated that lack of time, limited access to ultrasound machines, competing priorities and doubts about the added value of LUS were the main barriers. Time constraints are a well-known obstacle to POCUS training,^13 21 22^ and perceived clinical benefits of LUS appear to be key drivers of engagement. Successful certification therefore depends not only on training content and number of supervised LUS examinations but also on individual motivation and institutional support.

Most participants reported feeling comfortable acquiring LUS images after 6–15 supervised examinations, in line with previous evidence that basic proficiency can be achieved after 5–25 supervised scans.^12 13 19 26 27^ The requirement of five supervised examinations was generally perceived as appropriate, supporting the feasibility of the PLUS-IS-LESS assessment structure. However, sustained proficiency likely requires longitudinal reinforcement through periodic supervised practice and structured feedback.^21 22^

Although this study was conducted in Swiss EDs, LUS is increasingly used across diverse healthcare settings, and many of the training and implementation challenges identified are likely relevant beyond this context. Nevertheless, adaptation to local organisational structures and workflows may be required when applying similar training programmes in other healthcare systems. National or regional frameworks for LUS training, assessment and certification, together with institutional support for protected training time and innovative organisational models such as centralised image review and tele-ultrasound support, may further promote harmonisation, quality assurance and sustainable LUS adoption.^28–30^

This study has several strengths. It is the first to evaluate a standardised LUS training and certification programme across multiple Swiss EDs. The use of a structured, objective assessment method adapted from the LUS-OSAUS framework enabled robust evaluation of both theoretical knowledge and practical skills; the multicentre design enhances external validity and demonstrated the feasibility of remote supervision in routine ED practice.

Several limitations must be acknowledged. The final sample size (N=83) was sufficient to describe overall performance but may have been underpowered to detect subgroup differences, and centre-level comparisons were limited by small numbers. Selection bias cannot be excluded, as clinicians with greater interest in LUS may have been more likely to engage with the training programme. In addition, the total number of eligible physicians and characteristics of non-participants were not systematically recorded, limiting comparison between participants and non-participants. The certification threshold (≥80% per examination) was based on expert consensus rather than empirically validated cut-off values, which may limit the validity and comparability of the outcome definition and may have masked item-level weaknesses. However, this pragmatic threshold reflects commonly used standards in competency-based medical education, although no universally validated cut-off exists for LUS proficiency. Finally, long-term retention and clinical impact of LUS skills were not assessed and would require longitudinal follow-up.

Despite these limitations, this study adds to the POCUS education literature by supporting the feasibility of structured, competency-based LUS training and certification in emergency medicine and by identifying key educational and organisational determinants of long-term success.

## Conclusion

This study shows that a concise, structured LUS training and certification programme can improve theoretical knowledge and image acquisition skills among ED physicians. However, despite structured training and focused proficiency testing, only half of participants achieved certification. Among those who completed certification, nearly three-quarters achieved the predefined proficiency criteria, supporting the feasibility and educational impact of this approach in routine emergency care. This supports the educational value of the programme when fully undertaken but highlights the challenges of broad LUS implementation. Beyond technical and logistical barriers such as time constraints, limited access to ultrasound machines and competing clinical demands, integrating LUS findings into clinical decision-making remained difficult, and some participants perceived limited added clinical value of LUS in routine care. These suggest that successful implementation of LUS requires not only technical training but also ongoing supervision, clinical mentorship and support to strengthen diagnostic interpretation and bedside decision-making. Overall, this study provides valuable insights into barriers and facilitators and offers a practical framework for implementing LUS training programmes in emergency medicine and other clinical settings.

## Supplementary material

10.1136/bmjopen-2026-116508 online supplemental appendix 1

## Data Availability

Data are available upon reasonable request.
