# Supplementary material for: Proficiency in lung ultrasound and perceived barriers to adoption among emergency physicians after a short pneumonia training programme: a multicentre observational study in Swiss emergency department
Source: BMJ Open. 2026 Sep 8;16(9):e116508. doi: 10.1136/bmjopen-2026-116508 (PMC13561006; doi:10.1136/bmjopen-2026-116508)
Supplement: Supplementary file 1 [file bmjopen-2026-116508.reviewer_comments.pdf]

## PEER REVIEW HISTORY

BMJ Open publishes all reviews undertaken for accepted manuscripts. Reviewers are asked to complete a checklist review form and are provided with free text boxes to elaborate on their assessment. These free text comments are reproduced below.

### ARTICLE DETAILS

#### Title (Provisional)

Proficiency in lung ultrasound and perceived barriers to adoption among emergency physicians after a short pneumonia training program: a multicentre observational study in Swiss emergency department

#### Authors

Bessat, Cécile; Erard, Alizé; Roux, Alexia; Brahier, Thomas; Suttels, Veronique; Bingisser , Roland; Schwendinger, Markus; Bulaty, Tim; Fournier, Yvan; Della Santa, Vincent; Pfeil, Magali; Schwab, Dominique; Leuppi, Jörg D.; Geigy, Nicolas; Steuer, Stephan; Roos, Friedemann; Christ, Michael; Sirova, Adriana; Atzl, Alexandra; Napieralski, Fabian; Tagan, Damien; Albricht, Werner C.; Hugli, Olivier; Boillat-Blanco, Noémie; Garcia, Elena

---

### VERSION 1 - REVIEW

---

| | |
|---|---|
| **Reviewer** | **1** |
| **Name** | **van de Maat, Josephine** |
| **Affiliation** | **Radboudumc, Infectious diseases and global health** |
| **Date** | **16-Mar-2026** |
| **COI** | **None** |

---

I would like to thank the authors for this very important work. See my specific comments below.

Originality - does the work add enough to what is already in the published literature? If so, what does it add?

- Yes, this is an important addition to the available literature. Many studies focus on the diagnostic value of LUS in an experimental setting, but in order to actually improve quality of care the implementation process is crucial. That is what this paper nicely describes, including the challenges that arise, which is useful for all setting that want to implement LUS in their clinical practice.

Importance of work to readers - does this work matter to researchers, clinicians, patients, teachers, or policymakers?

- Yes, it is important for clinicians and researchers in the first place, since it helps to define further research questions (especially implementation research) and to define bottle necks for improving clinical care.

Evidence-based medicine - is the evidence presented scientifically and reliable?

- Yes

Research question - is it clearly defined and appropriately answered?

- Yes

Study design - is the overall design adequate?

- Yes

Participants - are they adequately described and their conditions well defined?

- There is a risk of selection bias towards participants that attended the training after invitation, which are probably the ones already more interested in LUS than the overall population of clinicians. How many were invited and how many attended? What were their characteristics?

- Medical supervisors were invited; are those the ones that in practice would actually perform the LUS? What is the role of junior doctors, and why were they not invited? In many settings junior doctors would do the first work-up (including imaging) of the patient, so training them may have more impact on the ED workflow.

- It should be clarified that participants are all ED physicians, right? This is not clear from the methods section, as also other consultants may work on ED's and could perform LUS (e.g. internal medicine consultants, paediatricians etc).

Methods - are they adequately described? Are they ethical?

- Page 5, line 34: semiology is an uncommon term, please define otherwise

- Practical question: how did the authors manage to plan a practical training of 1 hour on patients with pneumonia? Or did they only have to interpret images of patients?

- Otherwise clearly described with helpful figures

- Page 7, line 34: 6-12 months is a rather big difference to re-evaluate. Wat was the general follow-up interval? Any differences in results between participants that had 6 or 12 months follow-up?

- Page 7, line 58: why was the cut-off of 40 years chosen? And why would differences between german/French speaking regions be useful? Is there a difference in workflow/training otherwise? Were there difference between registrars and consultants? Since they probably have a different role in the workflow of managing the patient.

-

Results - do they answer the research question? Are they credible and well-presented?

Interpretation and conclusions - are they warranted by and sufficiently derived from/focused on the data? Is the message clear?

- In the results section only the participants that finished the quiz and LUS examinations were included in the analysis, while in the methods section was stated that all participants enrolled in the program would be included. This should be clarified better, be consistent in the definition of participants/study population.

- Page 8, line 27: add n=61 to the 50%, otherwise it is unclear if it is 50% of the 122 or of the 83 mentioned earlier. The figure is clear though. IN the figure it would be helpful to add the number of invited physicians, to get an idea of inclusion rate and potential selection bias.

- Table 1: as mentioned before, it may be useful to report the registrars vs senior physicians separately

- Page 12, line 25: how is patient weakness a difficulty? Meaning that the patient is too severely ill to perform this additional diagnostic test? Please clarify.

- The difficulties mentioned are very different, it would be useful to distinguish patient factors / technical factors / resources / workflow etc.

- Discussion/conclusion: the conclusion only mentions modifiable and technical barriers, and is quite positive about the training program and its results. However, one of the main challenges is also in the decision-making process: participants score low on the clinical conclusion/diagnosis based on LUS, and mention to see little added value of LUS in clinical care as a reason not to use LUS anymore after the training. This should be elaborated on more in the discussion and the conclusion. Even when there would be high success rates of the training, these more complex decision-making factors would still hamper the improvement of clinical care. This is better described in the conclusion of the abstract, but not of the full paper.

References - are they up to date and relevant? Any omissions?

- No comments

Abstract/summary/key messages - do they reflect accurately what the paper says?

- Line 29 unclear sentence: which characteristics?

- Line 28: success rate is not clear enough from the abstract. Add definition of success.

- Line 42: Add ‘after’ before first sentence?

- Please align conclusion of the abstract with the conclusion of the full paper, see comments there.

- Strengths and limitations should also be mentioned in the discussion of the paper. And another limitation is the unclarity about the number of physicians that were invited, see comment on selection bias in the methods section.

---

| | |
|---|---|
| **Reviewer** | **2** |
| **Name** | **Fu, Pin-Kuei** |
| **Affiliation** | **Taichung Veterans General Hospital** |
| **Date** | **17-Mar-2026** |
| **COI** | **None** |

---

This manuscript addresses a clinically relevant and timely question regarding the effectiveness and scalability of short-course lung ultrasound training in ER physicians. It provides valuable insights into both competency acquisition and barriers to implementation, particularly highlighting the gap between technical proficiency and clinical integration.

The study is strengthened by its multicentre design, embedding within a pragmatic trial, and the use of a structured assessment framework (adapted LUS-OSAUS). The combination of theoretical, practical, and follow-up survey data enhances the robustness of the findings. Importantly, the study demonstrates that while technical skills can be acquired through short training, translating these skills into clinical decision-making remains challenging.

##Major Comments

1. Validity of certification criteria

The certification threshold (≥80%) is based on expert consensus rather than empirical validation. This raises concerns regarding the validity and reproducibility of the outcome definition. The authors should provide a stronger justification or acknowledge this limitation more explicitly.

2. Selection bias due to incomplete participation

Approximately one-third of participants did not complete the full assessment pathway. This may introduce selection bias and potentially overestimate the reported proficiency rates. A comparison between completers and non-completers is recommended.

3. Lack of clinical outcome assessment

The study focuses on training outcomes but does not evaluate clinical endpoints such as diagnostic accuracy, patient outcomes, or antibiotic stewardship impact. This limits the ability to determine the true clinical significance of the intervention.

4. Absence of longitudinal follow-up

The study does not assess long-term retention of LUS skills. Given the known issue of skill decay in POCUS training, this represents an important limitation that should be addressed or discussed.

5. Limited statistical approach

The analysis relies primarily on univariate methods. Incorporating multivariable regression models would strengthen the identification of independent predictors of certification and reduce confounding.

6. Lack of implementation science framework

The manuscript would benefit from integration of established frameworks (e.g., RE-AIM, CFIR) to better interpret barriers to adoption and move beyond descriptive findings.

6. Insufficient exploration of clinical integration gap

While the study identifies difficulty in translating LUS findings into clinical decision-making, this key issue is not explored in depth. Further discussion on cognitive, workflow, and system-level factors is warranted.

##Minor Comments

1. Clarification of outcomes

The distinction between primary and secondary outcomes should be clearly defined.

2. Objective formulation

The study objectives are well described but remain descriptive. The inclusion of explicit hypotheses or conceptual pathways would strengthen the manuscript.

3. Participant characteristics

Additional detail regarding baseline LUS experience and prior training would improve interpretation of results.

4. Age-related findings

The association between younger age and higher certification rates should be interpreted cautiously, as potential confounders are not adjusted for.

5. Barrier analysis

The discussion of barriers (e.g., time, equipment, perceived value) could be expanded to better reflect their interaction with ED workflow and institutional constraints.

6. Generalizability

The applicability of findings to other healthcare systems should be discussed, given the Swiss setting.

7. Policy implications

Expanding on system-level and policy implications would enhance the manuscript's impact.

Overall, this is a well-conducted and practically relevant study that highlights an important gap between training and implementation. With further refinement of methodological rigor and deeper conceptual framing, the manuscript has the potential to make a meaningful contribution to the field of POCUS education and clinical integration.

---

## VERSION 1 - AUTHOR RESPONSE

<table>
<tr><td colspan="2">Reviewer: 1<br>Dr. Josephine van de Maat, Radboudumc, Erasmus MC Sophia Children Hospital<br>Comments to the Author:</td></tr>
<tr><td colspan="2">Participants - are they adequately described and their conditions well defined?</td></tr>
<tr><td>- There is a risk of selection bias towards participants that attended the training after invitation, which are probably the ones already more interested in LUS than the overall population of clinicians. How many were invited and how many attended? What were their characteristics?</td><td>Thank you for this important comment. We acknowledge the potential risk of selection bias, as clinicians with greater pre-existing interest in LUS may have been more likely to engage with the training program.<br><br>However, the PLUS-IS-LESS LUS training program was designed to target all medical supervisors (senior registrars and senior physicians) in participating emergency departments, rather than relying on voluntary recruitment of selected individuals. Physicians unable to attend the initial training session were offered catch-up sessions.<br><br>Characteristics of participating physicians are described in table 1. Unfortunately, the total number of eligible physicians invited across centres was not systematically recorded, and characteristics of clinicians who did not attend were not collected.<br><br>We have clarified the recruitment process in the manuscript in the Methods section and acknowledge this as a limitation of the study in the Discussion section.<br><br>See in Methods: Page 5; line 148-153<br><br>And in Discussion: Page 15; line 370-374: “Selection bias cannot be excluded, as clinicians with greater interest in LUS may have been more likely to engage with the training program. In addition, the total number of eligible physicians and characteristics of non-participants were not systematically recorded, limiting comparison between participants and non-participants.”</td></tr>
<tr><td>- Medical supervisors were invited; are those the ones that in practice would actually perform the LUS? What is the role of junior</td><td>Medical supervisors were selected because they were the clinicians primarily expected to perform LUS examinations in the participating</td></tr>
</table>

| | |
|---|---|
| doctors, and why were they not invited? In many settings junior doctors would do the first work-up (including imaging) of the patient, so training them may have more impact on the ED workflow. | emergency departments during the PLUS-IS-LESS study. Junior doctors were not included due to their frequent rotation between departments and institutions, which made systematic training and sustained participation throughout the study period logistically challenging and not feasible within the study framework. Training was therefore focused on senior staff to ensure continuity and consistency of intervention implementation across sites. |
| - It should be clarified that participants are all ED physicians, right? This is not clear from the methods section, as also other consultants may work on ED's and could perform LUS (e.g. internal medicine consultants, paediatricians etc). | We have clarified this in the method section.<br><br>Page 5; line 146-148: "The PLUS-IS-LESS LUS training program targeted all ED medical supervisors (senior registrars and senior physicians) from the participating EDs." |
| **Methods - are they adequately described? Are they ethical?** | |
| - Page 5, line 34: semiology is an uncommon term, please define otherwise | For more clarity we replaced this term appearing twice in the manuscript with the word "ultrasound findings" (page 6; line 165) and "ultrasound diagnostic patterns" (page 6; line 167). |
| - Practical question: how did the authors manage to plan a practical training of 1 hour on patients with pneumonia? Or did they only have to interpret images of patients? | For the practical training on patients with pneumonia, eligible patients were identified in hospital wards in the hours preceding each training session. These were patients already hospitalised with a confirmed diagnosis of pneumonia. Small groups of trainees then performed supervised bedside LUS examinations on these patients during the one-hour practical session. This allowed participants to acquire and interpret real patient images rather than relying solely on stored clips. |
| - Page 7, line 34: 6-12 months is a rather big difference to re-evaluate. Wat was the general follow-up interval? Any differences in results between participants that had 6 or 12 months follow-up? | Thank you for this comment. We had not written this very clearly in the manuscript. The follow-up survey was systematically sent 6 months after completion of the training program. The reported 6–12 months interval reflects variability in response time, as some participants took time and needed multiple reminders to completed the survey.<br><br>We have clarified this in the Methods section. Page 8; line 198-202: "Six months after their respective training program, ED physicians were invited to complete an online follow-up survey via the ProProfs® platform to assess barriers to completing the training and assessment process, provide feedback on the training format, and identify obstacles to the implementation of LUS in clinical workflows. In case of non-response, multiple reminders, up to twelve months after their respective training were sent to increase participation." |

| | |
|---|---|
| | Due to the limited sample size and the nature of the delay (response timing rather than planned follow-up), no formal comparison was performed between earlier and later respondents. |
| - Page 7, line 58: why was the cut-off of 40 years chosen? And why would differences between german/French speaking regions be useful? Is there a difference in workflow/training otherwise? Were there difference between registrars and consultants? Since they probably have a different role in the workflow of managing the patient. | The cutoff of 40 years was chosen because it reflects a clinically meaningful generational divide in Switzerland with respect to exposure to point-of-care ultrasound (POCUS). Physicians currently older than 40 completed most of their postgraduate training before the widespread introduction of POCUS in Switzerland (around 2010–2015), when ultrasound use in emergency care was still anecdotal. In contrast, physicians under 40 were in early or mid-training during the development and dissemination of POCUS, making systematic exposure and training more likely for this group.<br><br>Regarding the French and German speaking region, this comparison is relevant because ultrasound culture and training traditions differ markedly between linguistic regions in Switzerland. German-speaking centres have historically inherited a strong tradition of comprehensive ultrasound from Germany and Austria and integrated ultrasound into routine practice earlier. As a result, physicians in these centres generally adopted POCUS more rapidly and with fewer barriers. French-speaking regions, where comprehensive ultrasound by non-radiologist was less embedded historically, followed a different pathway of adoption. For these reasons, linguistic region represents a meaningful contextual variable for POCUS implementation.<br><br>In our participating centres, both registrars and consultants act as clinical supervisors and therefore share the same role in performing and validating LUS, as well as in jointly integrating ultrasound findings with junior doctors into clinical decision-making. While consultants additionally handle certain administrative and patient-flow management responsibilities from which registrars are exempt, these differences do not affect their respective roles in the ultrasound workflow. For the purposes of this study, registrars and consultants therefore fulfil comparable functions with respect to LUS practice. |
| **Results - do they answer the research question? Are they credible and well-presented? Interpretation and conclusions - are they warranted by and sufficiently derived from/focused on the data? Is the message clear?** | |

| | |
|---|---|
| - In the results section only the participants that finished the quiz and LUS examinations were included in the analysis, while in the methods section was stated that all participants enrolled in the program would be included. This should be clarified better, be consistent in the definition of participants/study population. | We thank the reviewer for this comment. All participants who attended the training were indeed included in the study population and completed the questionnaire on training perceptions and barriers.<br>However, the analysis of LUS proficiency was purposefully restricted to those who completed the full certification process (written quiz and supervised practical LUS examination), which accounted for 68% of participants. We have clarified this distinction in the Methods and Results sections to ensure consistency in the definition of the study population and the subset used for proficiency analyses. |
| - Page 8, line 27: add n=61 to the 50%, otherwise it is unclear if it is 50% of the 122 or of the 83 mentioned earlier. The figure is clear though. IN the figure it would be helpful to add the number of invited physicians, to get an idea of inclusion rate and potential selection bias. | We added the N=61.<br><br>As explained above, unfortunately, the total number of eligible physicians invited across centres was not systematically recorded. |
| - Table 1: as mentioned before, it may be useful to report the registrars vs senior physicians separately | As we mentioned previously, we decided not to report registrars and consultants separately as they both act as clinical supervisors and therefore share the same role. But we report age categories and years of medical experience. |
| - Page 12, line 25: how is patient weakness a difficulty? Meaning that the patient is too severely ill to perform this additional diagnostic test? Please clarify. | Patient weakness refers here to situations in which patients are too frail to change position, for instance unable to sit upright or turn adequately. In such cases, performing a full posterior lung ultrasound may require additional personnel to safely mobilize the patient, making the examination more challenging in routine practice.<br><br>We added this sentence to clarify Page 12; line 287-290: “Patient-related difficulties included obesity (82%) and patient weakness or poor cooperation (68%), particularly when patients were too frail or agitated to sit up or change position, making posterior lung scanning more cumbersome and often requiring additional staff.” |
| - The difficulties mentioned are very different, it would be useful to distinguish patient factors / technical factors / resources / workflow etc. | In the revised manuscript, we have grouped these challenges into patient-related, technical, and organisational/workflow-related factors to improve clarity and interpretation. |
| - Discussion/conclusion: the conclusion only mentions modifiable and technical barriers, and is quite positive about the training program and its results. However, one of the main challenges is also in the decision-making process: participants score low on the clinical conclusion/diagnosis based on LUS, | We agree that the challenges identified in this study extend beyond modifiable technical and logistical barriers and include more complex difficulties related to integrating LUS findings into clinical decision-making and the perceived limited added value of LUS in routine care. In response, we have revised |

| | |
|---|---|
| and mention to see little added value of LUS in clinical care as a reason not to use LUS anymore after the training. This should be elaborated on more in the discussion and the conclusion. Even when there would be high success rates of the training, these more complex decision-making factors would still hamper the improvement of clinical care. This is better described in the conclusion of the abstract, but not of the full paper. | both the Discussion and the full manuscript conclusion to further elaborate on these findings and better reflect the importance of cognitive and clinical integration barriers, in alignment with the abstract conclusion. |
| **Abstract/summary/key messages - do they reflect accurately what the paper says?** | |
| - Line 29 unclear sentence: which characteristics? | We defined better the characteristics in this sentence Page 2; line 57-59: "Success rates were compared between physicians according to their characteristics (age, sex, medical experience, previous use of ultrasound or LUS, linguistic region of work, and type of hospital) using a chi-square test." |
| - Line 28: success rate is not clear enough from the abstract. Add definition of success. | We added this sentence: Page 2; line 56-57: "Success was defined as achieving a score of ≥80% on both the online quiz and supervised practical assessment." |
| - Line 42: Add 'after' before first sentence? | Ok done. |
| - Please align conclusion of the abstract with the conclusion of the full paper, see comments there. | We adapted the conclusion so they align better. |
| - Strengths and limitations should also be mentioned in the discussion of the paper. And another limitation is the unclarity about the number of physicians that were invited, see comment on selection bias in the methods section. | As mentioned above we added a paragraph with the strengths and limitations in the discussion section. |
| | |
| **Reviewer: 2**<br>**Dr. Pin-Kuei Fu, Taichung Veterans General Hospital**<br>**Comments to the Author:** | |
| ##Major Comments | |
| 1. Validity of certification criteria<br>The certification threshold (≥80%) is based on expert consensus rather than empirical validation. This raises concerns regarding the validity and reproducibility of the outcome definition. The authors should provide a stronger justification or acknowledge this limitation more explicitly. | We agree that the certification threshold, being based on expert consensus rather than empirical validation, may raise concerns regarding the validity and reproducibility of the outcome definition. We have revised the limitations section to make this point more explicit and to clarify that the threshold was defined pragmatically, in line with commonly used standards in competency-based medical education, although no universally validated cutoff exists for LUS proficiency.<br><br>See Page 15; line 374-379 "The certification threshold (≥80% per examination) was based on expert consensus rather than empirically validated cutoff values, which may limit the validity and comparability of the outcome definition and may have masked item-level weaknesses. However, this pragmatic threshold reflects commonly used standards |

| | |
|---|---|
| | in competency-based medical education, although no universally validated cutoff exists for LUS proficiency.” |
| 2. Selection bias due to incomplete participation<br>Approximately one-third of participants did not complete the full assessment pathway. This may introduce selection bias and potentially overestimate the reported proficiency rates. A comparison between completers and non-completers is recommended. | We agree that incomplete participation in the assessment pathway may introduce selection bias and potentially overestimate proficiency among physicians who completed the full certification process. To address this, we have reported both the overall certification rate among all trained physicians and the success rate among those completing the full assessment pathway. However, detailed comparative data between completers and non-completers were not systematically collected, precluding a formal comparison between these groups. Importantly, the 6-month follow-up survey was designed in part to help identify barriers and contextual factors that may explain why some participants did not complete the full certification process. |
| 3. Lack of clinical outcome assessment<br>The study focuses on training outcomes but does not evaluate clinical endpoints such as diagnostic accuracy, patient outcomes, or antibiotic stewardship impact. This limits the ability to determine the true clinical significance of the intervention. | Thank you for this comment. However, the aim of this embedded study was specifically to assess training outcomes and proficiency in lung ultrasound following a short training program and it was not designed to evaluate downstream clinical endpoints such as diagnostic accuracy, patient outcomes, or antibiotic stewardship impact. These clinical outcomes fall within the objectives of the main PLUS-IS-LESS trial and will be reported separately in the primary trial analysis. Furthermore, the lung ultrasound examinations used for practical assessment were performed on routine ward patients who were not enrolled in the PLUS-IS-LESS trial; therefore, no associated clinical outcome data were available for these examinations. |
| 4. Absence of longitudinal follow-up<br>The study does not assess long-term retention of LUS skills. Given the known issue of skill decay in POCUS training, this represents an important limitation that should be addressed or discussed. | We agree that long-term retention of LUS skills is an important consideration, particularly given the recognised risk of skill decay following point-of-care ultrasound training. Assessment of long-term skill retention was beyond the scope of the present substudy, which focused on immediate proficiency following completion of the short training program. We acknowledge that the absence of longitudinal follow-up limits conclusions regarding the durability of acquired competencies and have added this point to the Discussion as a study limitation. Page 15; line 379-380. |
| 5. Limited statistical approach<br>The analysis relies primarily on univariate methods. Incorporating multivariable regression models would strengthen the identification of independent predictors of certification and reduce confounding. | We agree that multivariable regression modelling could provide additional insight into independent predictors of certification and help account for potential confounding. However, given the relatively limited sample size and number of certification events in this |

| | |
|---|---|
| | observational substudy, we considered multivariable modelling at risk of overfitting and limited robustness. We therefore elected to use primarily univariate analyses to provide descriptive comparisons. |
| 6. Lack of implementation science framework<br>The manuscript would benefit from integration of established frameworks (e.g., RE-AIM, CFIR) to better interpret barriers to adoption and move beyond descriptive findings. | We agree that implementation science frameworks, such as RE-AIM or CFIR, may provide a useful structured approach for interpreting barriers to adoption and guiding implementation strategies. However, the present study was not originally designed as a formal implementation science study and was intended primarily as an observational assessment of LUS proficiency and perceived barriers following training. As such, barriers were explored descriptively rather than within a predefined implementation framework. We acknowledge that applying established implementation science frameworks could strengthen future investigations of LUS integration into emergency department workflows. |
| 6. Insufficient exploration of clinical integration gap<br>While the study identifies difficulty in translating LUS findings into clinical decision-making, this key issue is not explored in depth. Further discussion on cognitive, workflow, and system-level factors is warranted. | Thank you for this insightful comment. We definitely agree that difficulty translating LUS findings into clinical decision-making represents an important barrier extending beyond technical image acquisition and interpretation alone.<br>In the revised Discussion, we have expanded our interpretation of this finding to further address potential contributing factors, including cognitive challenges in integrating ultrasound findings into diagnostic reasoning, workflow constraints in busy emergency department settings, and broader system-level barriers such as limited supervision, competing clinical priorities, and local organisational practices. We agree that these factors are likely to play a key role in determining the real-world clinical impact of LUS implementation. |
| ##Minor Comments | |
| 1. Clarification of outcomes<br>The distinction between primary and secondary outcomes should be clearly defined. | We clarified distinction between primary and secondary objectives. Page 4-5; line 124-129. |
| 2. Objective formulation<br>The study objectives are well described but remain descriptive. The inclusion of explicit hypotheses or conceptual pathways would strengthen the manuscript. | Hypotheses were included at Page 5; line 129-132. |
| 3. Participant characteristics<br>Additional detail regarding baseline LUS experience and prior training would improve interpretation of results. | We agree that more detailed information regarding participants' baseline LUS experience and prior training would have provided additional context for interpreting the findings. However, no further information beyond the baseline characteristics already |

| | |
|---|---|
| | reported in the manuscript was collected systematically during the study. |
| 4. Age-related findings<br>The association between younger age and higher certification rates should be interpreted cautiously, as potential confounders are not adjusted for. | We agree that the association between younger age and higher certification rates should be interpreted cautiously, as analyses were based on univariate comparisons and potential confounding factors were not adjusted for. This finding is presented as exploratory, and no causal inference is intended. |
| 5. Barrier analysis<br>The discussion of barriers (e.g., time, equipment, perceived value) could be expanded to better reflect their interaction with ED workflow and institutional constraints. | In the revised Discussion, we have expanded this section to further elaborate on how time limitations, equipment availability, competing clinical priorities, and organisational factors may interact to affect the integration of LUS into routine emergency care. |
| 6. Generalizability<br>The applicability of findings to other healthcare systems should be discussed, given the Swiss setting. | We agree that the Swiss healthcare setting should be considered when interpreting the generalisability of our findings. However, lung ultrasound is increasingly used across a wide range of healthcare systems, and many of the practical and educational challenges identified in this study are likely relevant beyond the Swiss context. We have expanded the Discussion to acknowledge that, while implementation strategies may require adaptation to local organisational structures, the barriers and facilitators identified may be applicable to other emergency care settings.<br><br>We added this sentence in the discussion: "Although this study was conducted in Swiss EDs, LUS is increasingly used across diverse healthcare settings, and many of the training and implementation challenges identified are likely relevant beyond this context. Nevertheless, adaptation to local organisational structures and workflows may be required when applying similar training programs in other healthcare systems."<br><br>See page 15; line 355-359 |
| 7. Policy implications<br>Expanding on system-level and policy implications would enhance the manuscript's impact. | We had already mentioned in the discussion that national or regional frameworks for LUS training, assessment, and certification, would support sustainable LUS adoption. We expended this aspect in the revised discussion. |

---

## VERSION 2 - REVIEW

---

**Reviewer 2**

**Name** Fu, Pin-Kuei

**Affiliation** Taichung Veterans General Hospital


**Date** 16-Jul-2026

**COI**

---

The authors have adequately addressed the reviewers' comments and substantially improved the manuscript. The revisions clarify the study design, methodology, participant selection, and study limitations, while the Discussion has been strengthened by providing a more balanced interpretation of the findings and implementation challenges. Although several limitations remain, they have been appropriately acknowledged and do not compromise the overall validity of the study. This work provides valuable insights into lung ultrasound training and its implementation in emergency departments.
