# Supplementary material for: Proficiency in lung ultrasound and perceived barriers to adoption among emergency physicians after a short pneumonia training programme: a multicentre observational study in Swiss emergency department
Source: BMJ Open. 2026 Sep 8;16(9):e116508. doi: 10.1136/bmjopen-2026-116508 (PMC13561006; doi:10.1136/bmjopen-2026-116508)
Supplement: Supplementary file 2 [file bmjopen-16-9-s001.docx]

**APPENDIX**

**Table of contents**

[Table A1: Modifications of the LUS-OSAUS score to an adapted assessment score for Swiss emergency departments	2](#_Toc215570205)

[Table A2: Complete 32-questions online quiz adapted for Swiss emergency departments and based on the adapted LUS-OSAUS score	4](#_Toc215570206)

[Table A3: Follow-up survey	14](#_Toc215570207)

[Table A4: Certification rates by subgroups	20](#_Toc215570208)

[Table A5: Answers of the follow-up survey	21](#_Toc215570209)

Table A1: Modifications of the LUS-OSAUS score to an adapted assessment score for Swiss emergency departments

|  | Original LUS-OSAUS score | Adapted score for Swiss emergency departments |  |
| --- | --- | --- | --- |
| Area (n=6) | Item (n=17) | Item (n=18) | Comments |
| Indication |  |  |  |
|  | Evaluates the indication for lung ultrasound | Indication | We have grouped these two items into one item: “Indication” |
|  | Suggests focused questions that can be examined by lung ultrasound |  |  |
| Systematic lung ultrasound examination |  |  |  |
|  | Performs lung ultrasound systematically | Systematic lung ultrasound examination | We have grouped these two items into one item: “Systematic lung ultrasound examination” |
|  | Performs lung ultrasound on the basis of focused questions |  |  |
| Technical skills |  |  |  |
|  | Correct placement of patient |  | This item is not evaluated as we could not directly observe the exam (remote supervision) |
|  | Correct handling of the transducer |  | This item is not evaluated as we could not directly observe the exam (remote supervision) |
|  | Correct choice of transducer | Correct choice of probe |  |
|  |  | Correct choice of preset | added |
|  | Correct depth | Correct depth |  |
|  | Correct gain | Correct gain |  |
|  |  | Labelling anatomical position correctly | added |
|  |  | Saving images/videos correctly | added |
|  |  | Interpretability of the images | added |
| Findings |  |  |  |
|  | Correct assessment of pleura | Correct assessment of pleura |  |
|  | Correct assessment of B-line | Correct assessment of B-line |  |
|  | Correct assessment of consolidations | Correct assessment of consolidations |  |
|  | Correct assessment of pleural effusion | Correct assessment of pleural effusion |  |
|  |  | Correct assessment of diaphragm | added |
|  |  | Correct assessment of M-mode | added |
|  | Correct assessment of whether ultrasound-guided thoracentesis is safe | Correct assessment of whether ultrasound-guided thoracentesis is safe |  |
| Documentation |  |  |  |
|  | Documents findings in patient’s chart | Documents findings in patient’s chart |  |
| Conclusion |  |  |  |
|  | Able to make diagnosis on the basis of lung-ultrasound findings | Able to make diagnosis on the basis of lung-ultrasound findings |  |
|  | Able to integrate lung-ultrasound findings with patient’s history |  | This item is not evaluated |

Table A2: Complete 32-questions online quiz adapted for Swiss emergency departments and based on the adapted LUS-OSAUS score

Link to the quiz : <https://www.proprofs.com/quiz-school/ugc/story.php?title=plusisless-kssg-assessment-of-lung-ultrasound-skillsev>

| Questions | Image or video | Multiple choice |
| --- | --- | --- |
| Q1. This physician is examining a patient with dyspnea. What is he looking for when scanning the lateral inferior quadrant (4R)? |  | A. Pneumothorax  B. Consolidation  C. Pleural effusion  D. B and C are correct (correct)  E. All statements are correct |
| Q2. Which probe enables the deepest scanning? | A. B. | A. Linear probe  B. Convex probe (correct) |
| Q3. This physician examines a febrile man for suspected pneumonia. Which statement is true? |  | A. Lung ultrasound has the same performance as chest X-ray for the diagnosis of pneumonia  B. Lung ultrasound has low sensitivity and specificity for the detection of consolidation  C. Lung ultrasound can detect subpleural pneumonia, which represent 95% of pneumonias (correct) |
| Q4. When doing a lung ultrasound for suspected pneumonia, which quadrants should you scan? |  | A. Upper quadrants (1, 3, 5)  B. Lower quadrant that are where most of the pneumonia are located (2, 4, 6)  C. All quadrants (correct) |
| Q5. What is the best probe and preset to look for a pleural effusion? |  | A. Convex probe on the abdominal preset (correct)  B. Linear probe on the lung preset |
| Q6. What is the minimal amount of pleural effusion detectable on a lung ultrasound? |  | A. Around 1ml  B. Around 10 ml (correct)  C. Around 100 ml  D. Around 200ml |
| Q7. Which description best matches this video? |  | A. A-line pattern with normal lung sliding  B. B-line pattern with >= 3 B-lines  C. Small lung consolidation <1cm (correct)  D. Lung consolidation >1cm |
| Q8. This is an image of the right lateral quadrant (4R) in an "abdomen" preset: we observe the liver, the diaphragm and a large pleural effusion, note the thoracic spine sign. Click on the pleural effusion. |  |  |
| Q9. Which description best matches this video? |  | A. Lung consolidation >1cm with "hepatization"  B. Left pleural effusion  C. Curtain sign: the lung passes over the spleen during inspiration, visualized in an "abdomen" preset (correct)  D. B-line pattern with >= 3 B-lines |
| Q10. Which description best matches the image? |  | A. A-line pattern (dry lung or pneumothorax, impossible to distinguish without video) (correct)  B. A-line pattern (dry lung)  C. A-line pattern (pneumothorax)  D. A-line and B.line patterns |
| Q11. Click on the pleural line |  |  |
| Q12. Is lung sliding present? |  | A. Yes  B. No (correct) |
| Q13. Click on the B-line |  |  |
| Q14. B-lines on lung ultrasound (indicating interlobular septal thickening) have a very broad differential diagnosis (heart failure, interstitial lung disease, pneumonia...). |  | A. True (correct)  B. False |
| Q15. Is lung sliding present? |  | A. Yes (correct)  B. No |
| Q16. Click on the consolidation |  |  |
| Q17. Which description best matches this M-mode image? |  | A. Barcode sign (absence of lung sliding, as seen e.g. in pneumothorax) (correct)  B. Seashore sign (presence of lung sliding) |
| Q18. Which description best matches this video? |  | A. A-line pattern with normal lung sliding  B. A' profile with absent lung sliding  C. B-line pattern with >= 3 B-lines  D. Consolidation > 1cm (correct) |
| Q19. Which description best matches this video? |  | A. A-line pattern with normal lung sliding  B. B-line pattern with irregular pleural line (correct)  C. Consolidation > 1 cm  D. Pleural effusion |
| Q20. Visualization of the left lateral quadrant. We observe the spleen, the diaphragm and a large pleural effusion. Click on the spleen. |  |  |
| Q21. Which description best matches this video? |  | A. A-line pattern with normal lung sliding (correct)  B. Pleural effusion  C. B-line pattern (>= 3 B-lines per field)  D. Lung consolidation >1cm |
| Q22. Which description best matches this video? |  | A. A-line pattern with normal lung sliding  B. A' pattern with absent lung sliding, lung-point visualized  C. B-line pattern with >= 3 B-lines and a thickened pleural line (correct)  D. Major lung consolidation with hepatization |
| Q23. Which description best matches the image? |  | A. Large complex pleural effusion with plankton sign, difficult to drain  B. Large uncomplicated pleural effusion, easy to drain  C. Small pleural effusion, difficult to drain  D. Septated, complex pleural effusion, difficult to drain (correct) |
| Q24. Which description best matches this video? |  | A. A-line pattern with normal lung sliding  B. B-line pattern with >= 3 B-lines  C. Lung consolidation > 1 cm (correct)  D. Pleural effusion |
| Q25. Which description best matches this video? |  | A. A-line pattern with normal lung sliding  B. A' profile with absent lung sliding  C. Small lung consolidation <1cm and irregular pleural line (correct)  D. Pleural effusion |
| Q26. Which description best matches this video? |  | A. Poor quality image (evaluation of pleural effusion using the "lung" pre-set)  B. Small quantity pleural effusion, difficult to drain  C. Large pleural effusion, easy to drain (correct)  D. Pericardial effusion |
| Q27. Lung ultrasound is a point-of-care examination. However, it is essential that the results are well documented in the patient's chart. Which statement is correct? |  | A. It is necessary to document dated summary in the patient file, indicating the indication, clinical setting, ultrasound findings and overall interpretation of the lung US  B. Images should be saved in the patient file and accessible to other medical staff in charge  C. Correct documentation of results improves patient care and facilitates the work of other colleagues  D. All statements are correct (correct) |
| Q28. You examine a 69-year old patient with cough, fever and dyspnea for 4 days.  You perform a lung ultrasound and find the following images *(cf. video)* in posterior inferior, anterior inferior and lateral right quadrants. How do you describe your findings? |    | A. Ultrasound findings show a large complex pleural effusion, compatible with an empyema.  B. Ultrasound findings show a major consolidation, compatible with a right lobar pneumonia. (correct)  C. Ultrasound findings do not allow to confirm pneumonia, I ask for a chest X-ray. |
| Q29. You examine a 75-year-old patient known for hypertensive cardiomyopathy. He is consulting for acute dyspnea and cough.  You perform lung ultrasound and find the following images *(cf. photo)* over all the quadrants. How do you describe your findings? |    | A. Widespread B-line pattern which is suggestive of a panlobar pneumonia.  B. Widespread B-line pattern which is suggestive of pulmonary edema. (correct)  C. Irregular and thickened pleural line with small consolidations <1cm, which are aspecific findings |
| Q30. You examine a 55-year old patient with cough, acute chest pain and dyspnea for 12 hours. He is an active smoker.  You perform a lung ultrasound and find the following pattern over all quadrants *(cf video)*. How would you describe your findings? |      | A. A-line pattern which is suggestive of a healthy lung  B. A-line pattern with normal lung sliding. No evidence of pneumonia on lung US. Pulmonary embolism or COPD exacerbation could be a likely diagnosis. (correct)  C. A' pattern suggestive of pneumothorax  D. Widespread B-line pattern suggestive of pulmonary edema |
| Q31. You examine a healthy 45-year-old women with cough, fever and myalgias since 3 days.  You perform lung ultrasound and find an A-line pattern everywhere except when zooming on the left posterior quadrant (6L) *(cf video)*. What do you conclude? |    | A. Small consolidation <1cm, no pneumonia on lung US (correct)  B. Small consolidation <1cm, pneumonia on lung US  C. Consolidation >1cm, pneumonia on lung US |
| Q32. You examine a healthy 49-year-old man with cough and fever since 3 days.  You perform lung ultrasound and find the following image *(see left video)* on the left lung and superior quadrants of the right lung, but find the following image *(right video)* on the inferior quadrants of the right lung. How would you describe your findings? |    | A. A-line pattern on the left lung and basal right A' pattern compatible with a pneumothorax  B. Patchy B-line pattern compatible with a pneumonia  C. Focal B-line pattern compatible with a pneumonia (correct)  D. Small consolidation <1cm in the inferior quadrant of the right lung |

Table A3: Follow-up survey

Link to the survey : <https://www.proprofs.com/quiz-school/ugc/story.php?title=612-months-plusisless-assessment-of-lung-ultrasound-uptake-by-clinicians>

| Questions | Multiple choice |
| --- | --- |
| Q1: What is your gender? | A) Female B) Male |
| Q2: What is your age? | A) 25 – 29 years B) 30 – 39 years C) 40 – 49 years D) 50 – 59 years E) 60 – 69 years |
| Q3: What is your medical experience? | A) < 5 years B) 5 – 9 years C) 10 – 14 years D) 15 years or more |
| Q4: **Before** the PLUS-IS-LESS training course what was your experience with ultrasound (NOT specifically lung ultrasound)? | A) I had never performed ultrasound. B) I had performed only a few ultrasounds. C) I already used ultrasound in my daily practice D) I already was a certified ultrasonographer |
| Q5: **Before** the PLUS-IS-LESS training course what was your experience with **lung** ultrasound ? | A) I had never performed lung ultrasound B) I had performed only a few lung ultrasounds C) I already used lung ultrasound in my daily practice D) I already was a certified lung ultrasonographer |
| Q6: Have you taken any other ultrasound course since the PLUS-IS-LESS course ? | A) Yes (🡪 Q7) B) No (🡪 Q8) |
| Q7: Which ultrasound course have you taken since the PLUS IS LESS course ? | *« Type here »* |
| Q8: Are you certified ? (Certified = took the PLUS-IS-LESS ultrasound course + passed the quiz + passed 5 lung ultrasounds) | A) Yes (🡪 Q10) B) No (🡪 Q9) |
| Q9: Why did you not obtain certification? (multiple answers possible) | A) I did not have enough time B) The ultrasound machine was often unavailable C) I had technical problems with the ultrasound machine (labelling, registration, exporting) D) I do not see the added value of ultrasound in my clinical practice E) I do not use ultrasound enough in my clinical practice F) Lack of confidence or fear of missing the diagnosis G) I misunderstood the certification requirements H) The certification requirements were too demanding I) I did not have enough onsite direct supervision J) None of the above |
| Q10: Do you think the online post training quiz was an appropriate way of assessing your theoretical knowledge on lung ultrasound ? | A) It was entirely appropriate to evaluate my theoretical knowledge on lung ultrasound (🡪 Q12) B) It was largely appropriate to evaluate my theoretical knowledge on lung ultrasound (🡪 Q12) C) It was neutral or unclear in its appropriateness to evaluate my theoretical knowledge on lung ultrasound (🡪 Q11) D) It was mostly inappropriate to evaluate my theoretical knowledge on lung ultrasound (🡪 Q11) E) It was completely inappropriate to evaluate my theoretical knowledge on lung ultrasound (🡪 Q11) |
| Q11: Why do you think the quiz was partially appropriate or inappropriate? What would you have changed? | *« Type here »* |
| Q12: What did you think about the distant supervision of 5 lung ultrasound ? (= practical certification requirement) | A) I was comfortable with distant supervision B) I was comfortable with distant supervision although I would have preferred onsite direct supervision C) I don’t have any preference between online or onsite supervision D) I was not entirely comfortable with online distant supervision E) I was not comfortable with online distant supervision |
| Q13: What did you think about the amount of 5 lung ultrasounds to obtain certification for the study ? | A) That was too much (🡪 Q14) B) That was slighty excessive (🡪 Q14) C) That was the right amount (🡪 Q15) D) That was somewhat insufficient (🡪 Q14) E) That was not enough (🡪 Q14) |
| Q14: How many lung ultrasounds should have been required and why? | *« Type here »* |
| Q15: How often do you use lung ultrasound in your clinical practice ? | A) Everyday B) Several times a week C) Once a week D) 2-3x per month E) Once a month F) Less than once a month G) Never |
| Q16: After how many lung ultrasound did you feel comfortable in recording good quality images/videos? | A) 1 – 5 B) 6 – 10 C) 11 – 15 D) 16 – 30 E) 31 – 50 F) > 50 |
| Q17: For what indications do you usually perform lung ultrasound ? (multiple answers possible) | A) Suspicion of pneumothorax B) Suspicion of pneumonia C) Suspicion of pulmonary effusion D) Suspicion of atelectasis E) Suspicion of pulmonary oedema (any ethiology) F) Suspicion of pulmonary embolism G) Suspicion of asthma / COPD exacerbation H) As a locator prior to a pleural puncture |
| Q18: What pathologies do you feel comfortable diagnosing/excluding with lung ultrasound ? (multiple answers possible) | A) Pneumothorax B) Pneumonia C) Pulmonary effusion D) Atelectasis E) Pulmonary oedema (any ethiology) F) Pulmonary embolism G) Asthma / COPD exacerbation H) None of the above |
| Q19: What facilitates your interpretation of lung ultrasound ? (multiple answers possible) | A) My technique in ultrasound B) The quality of my image C) My experience in ultrasound D) My knowledge prior to the PLUS IS LESS ultrasound course E) My knowledge obtained during the PLUS IS LESS ultrasound course F) The patient's clinical presentation G) Sharing the images with onsite colleagues H) Sharing the images on online chats I) None of the above |
| Q20: Do other factors facilitate your interpretation of ultrasound ? | *« Type here »* |
| Q21: What "patient-related" difficulties do you frequently encounter during lung ultrasound ? (multiple answers possible) | A) Breasts impeding good image acquisition B) Obesity impeding good image acquisition C) Poor patient cooperation (for instance because of confusion/agitation) D) Patient weakness, hard to mobilise E) Underlying pulmonary pathology (e.g. pulmonary fibrosis) impeding image interpretation F) None of the above |
| Q22: Have you encounter any other patient-related difficulties ? | *« Type here »* |
| Q23: What "technical-related" difficulties do you frequently encounter? (multiple answers possible) | A) Unavailability of the ultrasound machine B) Lack of time (difficult integration into emergency flow) C) Lack of supervision D) Lack of confidence in my technique E) Difficulty in handling the ultrasound machine interface (labelling, registration) F) Difficulty in finding an adequate environment to do the ultrasound (for instance not in the hallway) G) Disinfection of the ultrasound machine H) None of the above |
| Q24: Have you encounter any other technical-related difficulties ? | *« Type here »* |
| Q25: What "examination-related" difficulties do you frequently encounter ? | A) Positioning the probe in the correct quadrant B) Finding the curtain sign (quadrant 4 = lateral inferior ) C) Finding the spine (quadrant 4 = lateral inferior) D) The heart not allowing to check accurately the quadrant L2 = left antero-inferior E) Finding the diaphragm F) Positioning the patient for the posterior's quadrants G) Difficulty differentiating L3 from L4 and R3 from R4 (latero-superior from latero-inferior) H) None of the above |
| Q26: Have you encounter any other examination-related difficulties ? | *« Type here »* |
| Q27: What kind of continuing education training would you have liked to have after the PLUS IS LESS training course ? (multiple answers possible) | A) More E-learning B) Frequent theoretical refresh on site C) Multiple choice questions to test knowledge D) Interactive chat for ultrasound related questions E) Pathological image sharing F) More distant online supervised ultrasound G) More onsite supervised ultrasound H) Dedicated time for practical training |
| Q28: Do you have any other comments ? | *« Type here »* |

Table A4: Certification rates by subgroups

| Category | Subgroup | Success rate (95% CI) | p-value |
| --- | --- | --- | --- |
| Age |  |  | p = 0.009 |
|  | < 40 years | 0.85 (0.73 – 0.93) |  |
|  | ≥ 40 years | 0.57 (0.41 – 0.72) |  |
| Gender |  |  | p = 0.967 |
|  | Female | 0.71 (0.53 – 0.85) |  |
|  | Male | 0.75 (0.62 – 0.84) |  |
| Medical experience |  |  | p = 0.128 |
|  | < 15 years | 0.84 (0.68 – 0.93) |  |
|  | ≥ 15 years | 0.67 (0.53 – 0.78) |  |
| Ultrasound experience |  |  | p = 1 |
|  | Non-regular user | 0.71 (0.45 – 0.88) |  |
|  | Regular user | 0.74 (0.62 – 0.83) |  |
| Lung ultrasound experience |  |  | p = 1 |
|  | Non-regular user | 0.73 (0.58 – 0.84) |  |
|  | Regular user | 0.74 (0.59 – 0.85) |  |
| Language region |  |  | p = 0.583 |
|  | French-speaking | 0.70 (0.55 – 0.81) |  |
|  | German-speaking | 0.78 (0.62 – 0.88) |  |
| Hospital type |  |  | p = 1 |
|  | Secondary | 0.73 (0.57 – 0.84) |  |
|  | Tertiary | 0.74 (0.60 – 0.85) |  |

Table A5: Answers of the follow-up survey

| Use of LUS |  | N = 81 | % |
| --- | --- | --- | --- |
|  | Less than once a month | 2 | 2.5 |
|  | Once a month | 2 | 2.5 |
|  | 2-3x per month | 4 | 4.9 |
|  | Once a week | 25 | 30.9 |
|  | Several times a week | 22 | 27.2 |
|  | Everyday | 26 | 32.1 |
| Amount of LUS to feel comfortable |  | N = 81 | % |
|  | 1 - 5 | 15 | 18.5 |
|  | 6 - 10 | 22 | 27.2 |
|  | 11 - 15 | 27 | 33.3 |
|  | 16 - 30 | 12 | 14.8 |
|  | 31 - 50 | 2 | 2.5 |
|  | > 50 | 3 | 3.7 |
| Amount of 2 or 5 LUS to obtain certification for the study |  | N = 81 | % |
|  | That was too much | 1 | 1.2 |
|  | That was slightly excessive | 8 | 9.9 |
|  | That was the right amount | 54 | 66.7 |
|  | That was somewhat insufficient | 16 | 19.8 |
|  | That was not enough | 2 | 2.5 |
| Cause of non-certification |  | N = 16 | % |
|  | I did not have enough time | 13 | 81.2 |
|  | The ultrasound machine was often unavailable | 16 | 100 |
|  | I had technical problems with the ultrasound machine (labelling, registration, exporting) | 1 | 6.2 |
|  | I do not see the added value of ultrasound in my clinical practice | 16 | 100 |
|  | I do not use ultrasound enough in my clinical practice | 2 | 12.5 |
|  | Lack of confidence or fear of missing the diagnosis | 4 | 25 |
|  | I misunderstood the certification requirements | 16 | 100 |
|  | The certification requirements were too demanding | 2 | 12.5 |
|  | I did not have enough onsite direct supervision | 2 | 12.5 |
|  | None of the above | 2 | 12.5 |
| Technical related difficulties |  | N = 81 | % |
|  | Unavailability of the ultrasound machine | 22 | 27.2 |
|  | Lack of time (difficult integration into emergency flow) | 62 | 76.5 |
|  | Lack of supervision | 16 | 19.8 |
|  | Lack of confidence in my technique | 19 | 23.5 |
|  | Difficulty in handling the ultrasound machine interface (labelling, registration) | 14 | 17.3 |
|  | Difficulty in finding an adequate environment to do the ultrasound (for instance not in the hallway) | 16 | 19.8 |
|  | Disinfection of the ultrasound machine | 1 | 1.2 |
|  | None of the above | 13 | 16.0 |
| Patient related difficulties |  | N = 81 | % |
|  | Breasts impeding good image acquisition | 35 | 43.2 |
|  | Obesity impeding good image acquisition | 66 | 81.5 |
|  | Poor patient cooperation (for instance because of confusion/agitation) | 53 | 65.4 |
|  | Patient weakness, hard to mobilise | 55 | 67.9 |
|  | Underlying pulmonary pathology (e.g. pulmonary fibrosis) impeding image interpretation | 33 | 40.7 |
|  | None of the above | 3 | 3.7 |
| Examination related difficulties |  | N = 81 | % |
|  | Positioning the probe in the correct quadrant | 6 | 7.4 |
|  | Finding the curtain sign (quadrant 4 lateral inferior) | 6 | 7.4 |
|  | Finding the spine (quadrant 4 lateral inferior) | 30 | 37.0 |
|  | The heart not allowing to check accurately the quadrant L2 left antero-inferior | 47 | 58.0 |
|  | Finding the diaphragm | 3 | 3.7 |
|  | Positioning the patient for the posteriors quadrants | 29 | 35.8 |
|  | Difficulty differentiating L3 from L4 and R3 from R4 (latero-superior from latero-inferior) | 10 | 12.3 |
|  | None of the above | 18 | 22.2 |
| Facilitate interpretation |  | N = 81 | % |
|  | My technique in ultrasound | 59 | 72.8 |
|  | The quality of my image | 65 | 80.2 |
|  | My experience in ultrasound | 68 | 84.0 |
|  | My knowledge prior to the PLUS IS LESS ultrasound course | 45 | 55.6 |
|  | My knowledge obtained during the PLUS IS LESS ultrasound course | 52 | 64.2 |
|  | The patient’s clinical presentation | 63 | 77.8 |
|  | Sharing images with onsite colleagues | 40 | 49.4 |
|  | Sharing images on online chats | 2 | 2.5 |
|  | None of the above | 1 | 1.2 |
| Diagnosing/excluding with LUS |  | N = 81 | % |
|  | Pneumothorax | 79 | 97.5 |
|  | Pneumonia | 50 | 61.7 |
|  | Pulmonary effusion | 74 | 91.4 |
|  | Atelectasis | 20 | 24.7 |
|  | Pulmonary oedema (any ethiology) | 63 | 77.8 |
|  | Pulmonary embolism | 9 | 11.1 |
|  | Asthma / COPD exacerbation | 14 | 17.3 |
|  | None of the above | 2 | 2.5 |
| Indication LUS |  | N = 81 | % |
|  | Suspicion of pneumothorax | 81 | 100 |
|  | Suspicion of pneumonia | 55 | 67.9 |
|  | Suspicion of pulmonary effusion | 72 | 88.9 |
|  | Suspicion of atelectasis | 18 | 22.2 |
|  | Suspicion of pulmonary oedema (any ethiology) | 63 | 77.8 |
|  | Suspicion of pulmonary embolism | 20 | 24.7 |
|  | Suspicion of asthma / COPD exacerbation | 19 | 23.5 |
|  | As a locator prior to a pleural puncture | 67 | 82.7 |
| Appropriate quiz to assess LUS knowledge | Overall | N = 81 | % |
|  | It was entirely appropriate to evaluate my theoretical knowledge on lung ultrasound | 21 | 25.9 |
|  | It was largely appropriate to evaluate my theoretical knowledge on lung ultrasound | 52 | 64.2 |
|  | It was mostly inappropriate to evaluate my theoretical knowledge on lung ultrasound | 1 | 1.2 |
|  | It was neutral or unclear in its appropriateness to evaluate my theoretical knowledge on lung ultrasound | 7 | 8.6 |
| Distant supervision |  | N = 81 | % |
|  | I don’t have any preference between online or onsite supervision | 9 | 11.1 |
|  | I was comfortable with distant supervision | 36 | 44.4 |
|  | I was comfortable with distant supervision although I would have preferred onsite direct supervision | 28 | 34.6 |
|  | I was not comfortable with online distant supervision | 1 | 1.2 |
|  | I was not entirely comfortable with online distant supervision | 7 | 8.6 |
| Continuing education |  | N = 81 | % |
|  | More E-learning | 16 | 19.8 |
|  | Frequent theoretical refresh on site | 20 | 24.7 |
|  | Multiple choice questions to test knowledge | 6 | 7.4 |
|  | Interactive chat for ultrasound related questions | 15 | 18.5 |
|  | Pathological image sharing | 48 | 59.3 |
|  | More distant online supervised ultrasound | 13 | 16.0 |
|  | More onsite supervised ultrasound | 36 | 44.4 |
|  | Dedicated time for practical training | 42 | 51.9 |
